# Copper nanocoils synthesized through solvothermal method

**DOI:** 10.1038/srep16879

**Published:** 2015-11-26

**Authors:** Yanjuan Liu, Xiaowei Liu, Yongjie Zhan, Haiming Fan, Yang Lu

**Affiliations:** 1 Institute of Photonics and Photon Technology, Northwest University, Xi’an, Shaanxi, 710069, China; 2 Department of Bioengineering, School of Chemical Engineering, Northwest University, Xi’an, Shaanxi, 710069, China; 3 Department of Mechanical and Biomedical Engineering, City University of Hong Kong, Kowloon, Hong Kong SAR, China; 4 Center of Super-Diamond and Advanced Films (COSDAF), City University of Hong Kong, Kowloon, Hong Kong SAR, China

## Abstract

Recently helical nanostructures such as nanosprings and nanocoils have drawn great interests in nanotechnology, due to their unique morphologies and physical properties, and they may be potential building blocks in sorts of electromechanical, magnetic, photoelectronic and plasmonic devices at micro/nanoscales. In this report, multi-turns copper nanocoils were synthesized through a modified solvothermal method, in which the mixture of water and N-methyl-2-pyrrolidone (NMP) were selected as reaction medium and copolymer poly(1-vinylpyrrolidone-co-vinyl acetate) (PVP/VA 64E) as reductant. In the liquid solution, nanosprings could be formed from relaxed nanocoils and demonstrated high elasticity. These nanocoils and nanosprings are of single crystalline structure, with the characteristics wire diameters ranging from tens to a few hundreds of nanometers and the ring/coil diameters mostly ~10–35 microns. Their growth and deformation mechanisms were then investigated and discussed along with that of previously reported single-turn copper nanorings. This work could be of importance for researchers working on synthesis and applications of novel 1-D helical nanomaterials and their functional devices.

Syntheses of novel helical nanostructures such as nanosprings and nanorings, due to interesting physical properties and potential applications of these materials, compose one small but important part in recent nanoscience. These curved or coiled structures can be used as potential building blocks in many nano-mechanical, biomedical, and photoelectronic and plasmonic devices^1^,^2^. For examples, driven by external fields, nanosprings with magnetic heads can be used as nano-propellers for drugs delivery and local probes in liquid environment^3^, broadband circular polarizer can be built up using gold helices as photonic metamaterial units^4^. Various methods have been reported to fabricate these fascinating nanostructures, either in bottom-up or top-down approaches. In those “direct routes”, by rotating the substrate in both polar and azimuthal directions, nanosprings and other zigzag structures can be fabricated in so called glancing angle deposition (GLAD) and other similar methods^5^,^6^,^7^,^8^,^9^,^10^. Combination of 3-D direct laser writing (DLW) and following physical vapor deposition (on written models) made the sizes and shapes of as-prepared nanosprings adjustable to satisfy the flexible requirements.3,4 furthermore, hard templates such as anodic aluminum oxide (AAO) channels and even spiral vessels of vascular plant were also chosen to guide the growths of nanosprings^11^,^12^. In “indirect routes”, tensions induced by crystal lattice mismatching on bi-layer and multi-layer hetero-interfaces can twist planar deposition layers to form new spiral structures in wet etching processes^13^,^14^,^15^. while spiral nanostructures can also be fabricated in high temperature chemical reactions and template-free vapor depositions (non-GLAD), such as the earlier works on zinc oxide nanosprings and nanorings by Z.L. Wang *et al.*^16^,^17^,^18^. Since then, nanorings and nanosprings of tin oxide, AlN, SiO2, InP and CNTs have also been synthesized^19^,^20^,^21^,^22^,^23^. Their spiral growths were mainly induced by electrostatic interaction between opposite charged neighboring surfaces or by dislocation existing in crystals. Last but not least, coiled nanostructures can also be prepared in liquid circumstances and the mechanisms are usually involved with bending nanowires in confined micro-environments, or minimizing of the surface energy of nanowires^24^,^25^,^26^,^27^,^28^. Besides inorganic spiral nanostructures, organic materials of spiral nanostructures were also synthesized, mainly based on molecular self-assembly^29^,^30^. However, generally speaking, liquid phase reactions or settlements for the fabrication of nanocoils or nanosprings have been rarely reported and more challenging, despite their advantages such as simple reaction routes and mild reaction conditions.

In this report, copper nanocoils were synthesized via an improved solvothermal reaction modified from our earlier works^31^,^32^, in which single crystalline copper nanorings (single-turn) with characteristic widths ~100–200 nm were synthesized in alkali hydrothermal system or solvothermal system (in mixture of distilled water and NMP). In the current method for preparing multi-turns nanocoils and other copper helical nanostructures, we introduced one special copolymer (PVP/VA 64E) to replace the PVA as used in earlier works, which was proved to play a key role in forming these novel multi-turns coiled copper nanostructures. In addition, to the best of our knowledge, the application of PVP/VA as reductant in solvothermal reactions has not been reported yet.

## Results

**Figure 1:**
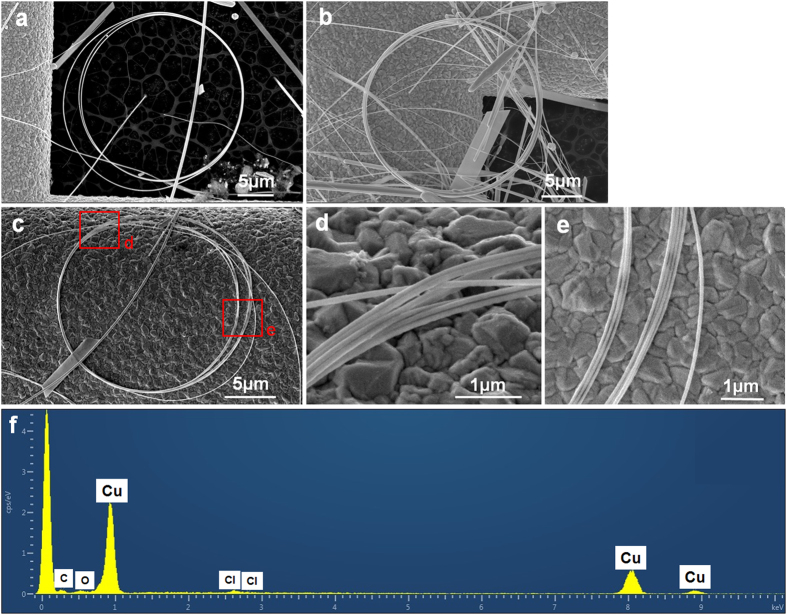
SEM images and EDS pattern of copper nanocoils. (**a–c**) Nanocoils with different numbers of turns. One nanocoil shown in (**c**) holds 8 turns and local fragments of which were shown in (**d**,**e**) at higher magnifications. (**f**) One EDS pattern of a typical nanocoil.

**Figure 2:**
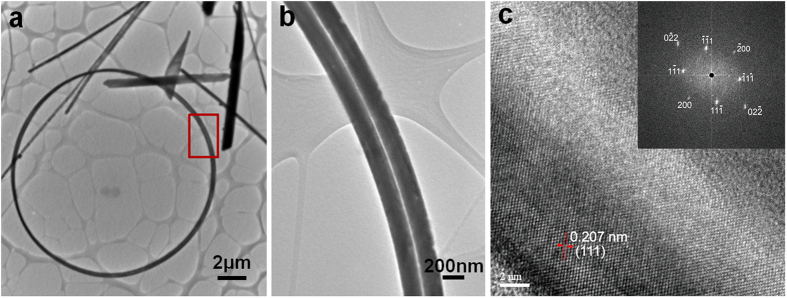
TEM images and SAED pattern of nanocoil. (**a**) Full view of one double-turns nanocoil and one section of which with parallel wires (**b**), corresponding section surrounded by red rectangle in (**a**); (**c**) multiple-point HRTEM imaging and SAED analysis, with a representative one shown in (**c**), indicates the single-crystallinity of the nanocoil samples.

**Figure 3:**
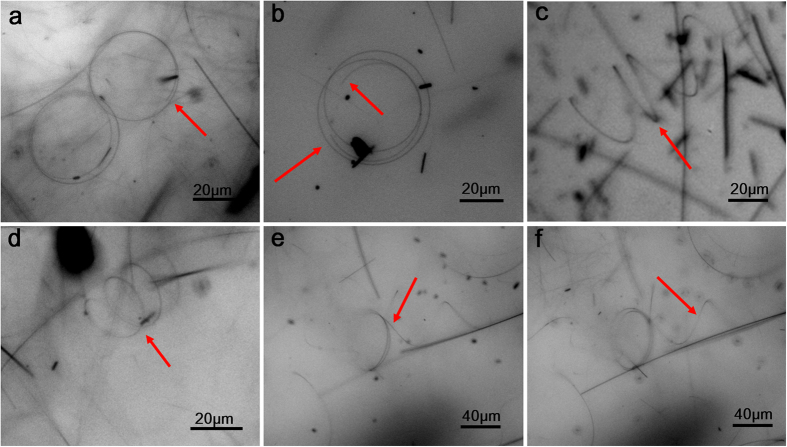
*In situ* morphologies of the nanocoils and its nanospring forms in the stock solution (optical images). (**a,b**) Nanocoils with non-closed ends (pointed by red arrows, similarly hereinafter); (**c,d**) nanosprings; (**e,f**) one double-turns nanocoil with a spiral tail, showing the stretching formation of a nanospring from a nanocoil in the solution.

Morphologies of as-prepared samples are shown in SEM images in Fig. 1. Along with conventional copper wires and plates of nano/submicron sizes, multi-turns nanocoils can be easily and clearly found in samples. These curved nanostructures compose a considerable proportion in the final product, roughly 15% or even higher among the as-fabricated nanostructures in the solutions. Diameters of these coils are mainly in range from 10 microns to 35 microns (see size distribution in Supplementary Information), and their line widths (characteristic diameters) are from about 90 nm up to about 200 nm. In nanocoils, bent copper wires of different turns sometimes adhere to each other and form sections of compact arrangements, which can attribute to the adhesive polymer coating on nanocoils, a typical by-product formed in similar hydrothermal syntheses through a so-called “synergistic soft-hard template mechanism” (SSHM)^33^,^34^. However diameters of different turns are not always uniform, which makes a complete compact arrangement around one coil impossible, so in nanocoils compact sections are accompanied by loose sections in many occasions. Careful checking the coiled nanowires in compact sections (Fig. 1d,e) shows that their arrangements can be in parallel or more compact way, do not strictly follow one single mode. Closed structure can be seen more clearly in nanocoils of fewer turns (Fig. S3, Supplementary information), and it can also be found there that not all the closed nanocoils have smoothly jointed ends. EDS pattern in Fig. 1f reveals that these nanocoils consist of copper (Cu) element, whereas the detected carbon (C) element should be mainly attributed to copolymer coating on the surface of the nanocoils. TEM analysis of nanocoils shown in Fig. 2(a,b) confirmed the near-perfect circular/multi-ring shape and uniform line widths of these nanocoils. Normally the typical line widths of nanocoils are too larger for suitable high resolution TEM imaging (HRTEM); only at edges of some thinner coils HRTEM images were made possible. Selected area electron diffraction (SAED) and localized HRTEM imaging on multiple locations along a single nanocoil was taken, such as an HRTEM image and the corresponding diffraction pattern calculated by fast Fourier transformation shown in Fig. 2c, to confirm the single-crystallinity of nanocoils (in <110> growth orientation along the wire axis). Furthermore, for nanocoils in the stock solution, *in situ* optical microscope observation was carried out, in which several nanocoils with non-closed ends were clearly shown in Fig. 3a,b. More interestingly, sometimes nanocoils can be relaxed and formed in multi-turn spring forms, *i.e.* nanosprings, as shown in Fig. 3c,d. Those nanosprings were from stretched or relaxed nanocoils in liquid environment, and such a stretching process has been evidenced in Fig. 3e,f as their intermediate state. These nanosprings demonstrated good elasticity and fatigue resistance under external mechanical disturbance (by shaking the solution or manipulated by a microprobe). However, due to the surface tension during the drying process for preparing free-standing SEM and TEM samples, different turns in nanosprings will tend to adhere to each other, becoming typical nanocoils shown in the EM images. These incompact coiled samples, and especially nanosprings in the stock solution, suggested that nanocoils are not simply bonded products of discrete single-turn nanorings, but indeed natural products of prolonged helical growth. A few other novel nanostructures, such as non-closed bent nanobelts can be also found in the solution (Fig. S4 and Fig. S5, Supplementary Information).

## Discussions

In this modified solvothermal method, the poly (1-vinyl pyrrolidone-co-vinyl acetate) (PVP/VA 64E) plays a critical role in preparing nanocoils and nanosprings. Compared with nanorings, to some extent the nanocoils can be simply defined as curved nanowires with far enlarged aspect ratios, however keeping coiled structure in prolonged growth process is not so easy. We usually get only two sorts of samples in early system using poly(vinyl acetate, PVA) as reductant: closed (single-turn) nanorings and straight nanowires or nanoplates, nanocoils and nanosprings cannot be found. The PVP part of this newly selected copolymer may help coiled nanowires to keep relative thinner line widths when they grow longer, which is a favorable factor in forming multi-turns structure. In non-solvothermal systems, Poly vinyl pyrrolidone (PVP) have been often used as guiding reagent^35^,^36^,^37^, its selective adherence to different crystal faces of metallic nanoparticles is in favor of the anisotropic growth of metal nanowires. In earlier experiments, it was found that introducing PVP of suitable amount in solvothermal system using PVA as reductant can decrease the linear widths of nanowires and correspondingly increase the proportion of curved copper nanowires in products under same reaction condition. Inspired by the integration effect of PVA and PVP in NMP/water system, Copolymer PVP/VA (64E) was selected and verified to be a suitable reductant and guiding reagent in synthesizing single/multi-turns nanorings, and nanosprings. (By the way, in experiments it was also found that this copolymer has no obvious effect in pure alkali hydrothermal system, it seems be effective mainly in NMP/water system.) The chemisorption of Br^–^ ions on different crystal faces of noble metals nanoparticles will alter the surface energies, and corresponding localized oxidative etching will lead to anisotropic growth too, to some extent the overall effect of which is similar to that of PVP, but Br^–^ ions is smaller than those polymer macromolecules, so especially at earlier growth stage when crystal seeds are rather small, bromides have their unique advantages in shape-controlling^37^,^38^. In experiments it was also found that the reaction times remarkably affect the morphologies and composition of different helical structures in copper samples. In samples under reactions times of 24 h to 48 h, nanocurves, nanocoils and nanosprings can be easily found; however in samples under longer reaction time (such as after one week), nanocoils and nanosprings with open ends will mostly disappear, as they are less stable compared to those end-closed nanorings and nanocoils. Based on the experiments, we can conclude the relationship between conditions of fabrication process and yields of nanocoils and nanocurves/nanowires as follow: Besides aforementioned factors like reaction time, PVP part in copolymer (PVP/VA) and Br^–^ are two key factors in our synthesis. They together help to produce the one-dimensional nanostructures with larger aspect ratios and relatively smaller line widths, which are favorable in forming multi-turns structures, whereas PVA alone can only produce single-turn nanorings or nanobelts. So the introducing of both PVP/VA and Br^–^ in solvothermal system will significantly increase the yield of multi-turns nanocoils. The yield of simple nanocurves (nanoarcs) is comparable with those of normal straight nanowires in many occasions, with most of thinner wires are nanocurves and thicker wires/belts are more straight ones–can be considered as re-stretched nanocurves with increased line widths upon prolonged reaction time.

In the initial growth stages, the bending or coiling growth of primary copper nanowires is possible and necessary, however the details of concrete driving forces is still unclear here. The as-prepared nanocoils/nanobelts can keep forming their helical structures for a long period of time even they are not end-closed ones, which may attribute to the fact that sliding planes are easily formed in single crystals of copper with relative low stacking fault energy. We suggested residual internal stresses in prepared samples that can affect their latter morphologies, the stresses help to stretch these closed structures out and provide them rather perfect circular shapes finally. One natural deduction therefore is that defects formed on these rings and coils can destroy their structure. We indeed investigated the deformation of unprotected copper nanorings in evaporating solution and justified above deduction. The aerugo defects in some occasions cause the nanocoils broken, with a tendency to stretch themselves out. Such an internal stress-induced deformation mode can partly explain the shapes of the nanobelts shown in Fig. S4 and Fig. S5 in Supplementary Information.

**Figure 4:**
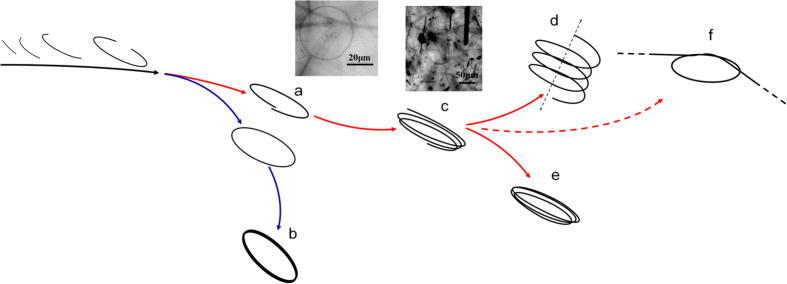
Scheme shows different evolutionary routes. The stages on red branches start from bent nanocurves with non-aligned ends (**a**), end in nanosprings (**d**) and closed nanocoils (**e**), inserted images from left to right show a bent nanocurve (**a**) and a multi-turns nanocoils (**c**) respectively. Nanospring (**d**) is formed from a stretched nanocoil (**c**). One possible branch towards fast-knot structure (**f**) is also shown; Stages on blue branch start from closed single-turn nanoring and end in widened nanobelt (**b**).

Therefore, the evolution routes in this solvothermal system can be derived and summarized in Fig. 4. At first some internal or external forces (induced by restricted growth circumstance, for example) bent (parts of) copper nanowires to form nanocurves (stage a) and nanorings (stage b), from nanocurves with misaligned opposite ends (stage a), multi-turns nanocoils and nanosprings (stages c and d respectively, along the red route in scheme, stages e represents grown nanocoils with closed ends) are synthesized sequentially. In long run, end-closed nanorings, nanobelts, nanocoils, and part of non-closed nanocoils with adhesive sections, can survive in a steady form. Unlike the case of ZnO nanorings^17^, elemental copper is isotropic material with non-polar surfaces, polarization-induced attractions between neighboring turns and closing ends cannot exist. Other helical structures prepared in liquid phase systems usually are results of template-guide or restrict^26^,^27^,^28^, those nanorings usually have diameters less than 100 nanometers, hindering their applications in certain areas, whereas copper nanocoils and nanosprings prepared in our method have much larger diameters of dozens up to hundreds of microns. Compared with products from other fabrication techniques (non-liquid phase methods, *e.g.* glancing angle deposition (GLAD) or microfabrication), our nanocoils have nearly perfect circular/helical and single crystalline structures, with better mechanical properties, especially elasticity^32^. In addition to Cu, our method can potentially be extended in preparing nanocoils of noble metals such as silver^35^, with better corrosion/ oxidation resistance, which could be very useful for environmental applications and surface enhanced Raman scattering (SERS) applications. Further research is needed to fully uncover the formation mechanisms and further enhance the yield and uniformity of the helical nanostructure products. Nevertheless, these metallic nanocoils and nanosprings should be unique and can be immediately useful for many functional nanodevice research. Their deformation in growth and corresponding evolution in lattice structure, their mechanic property measurement in liquid circumstance, their reaction to external electronic and magnetic fields are also interesting topics.

## Conclusion

In summary, this work demonstrates the use of specific copolymer, such as Poly(1-vinylpyrrolidone-co-vinyl acetate) (PVP/VA 64E), in rational synthesis of novel 1-D metallic nanostructures, in which multi-turns copper nanocoils have been successfully fabricated by a modified solvothermal method. These single crystalline nanocoils can be stretched or relaxed into nanospring forms in the solution, and demonstrated reasonably high elasticity and cyclic stability. We believe that these novel 1-D helical nanostructures could be ideal candidate for many functional applications such as plasmonic waveguides units in optoelectronic devices, surface enhanced Raman scattering (SERS) sensing, nano-mechanical and nano-electromechanical systems (NEMS) devices.

## Methods

### Solvothermal synthesis of copper nanocoils

In this work, CuCl and NaBr were standard, commercially available chemical compounds, N-methyl-2-pyrrolidone (NMP, M100588-2.5 L) was purchase from Aladdin Industrial Inc. Poly(1-vinylpyrrolidone-co-vinyl acetate) (PVP/VA 64E) was purchased from Shanghai Yuking Water Soluble Material Tech Co. Ltd. All reactants were of analytically pure and used as received without further purification. In the typical protocol, 0.45 g PVP/VA(64E) was firstly dissolved in 40 ml mixture of NMP and distilled water (V_NMP_:V_w_ = 2:1), then the solution was removed into a Teflon-lined stainless steel autoclave with a capacity of 50 mL. 0.1 g CuCl and 0.05 g NaBr were added in solution. After stirring the solution for 10 minutes the autoclave was sealed and heated in an oven of 220 °C for 24 h., and then naturally cooled down to room temperature. In dark brown solution deposition of reddish metallic luster could be found on the bottom of autoclave.

### Sample cleaning treatments

Treatment to get clean copper sample is suggested as follows: Clean test tube was firstly half-filled with NaCl solution, the density of which should be adjusted larger than that of the stock solution, and certain amount of ascorbic acid was also added in solution to provide a reducing environment. Along the inner wall of test tube stock solution that holding copper products was added slowly to cover the NaCl solution and form layered solution, the test tube was then kept static to let the copper samples fall across the interface and deposit on the bottom of test tube gradually, the total deposition process could last for one whole day. Then stock solution and NaCl solution was removed away and the copper sample on bottom was transferred into distilled water to wash away NaCl (and ascorbic acid), in the end little amount of hydrazine was added in solution to prevent copper deposition from corrosion. Copper samples can keep their morphologies for 1–2 months in such a circumstance. Picture of the treatment (Fig. S1) and corresponding discussion can be seen in Supplementary information.

### Microstructure characterizations

Optical images and videos were taken on three microscopes: Nikon Eclipse LV100, Qioptiq A-Zoom, and Phoenix XSP-30 equipped with a MC-D500U digital electric eye lens. More detailed morphology and structure information was revealed by scanning electron microscopy (SEM) and transmission electron microscopy (TEM). The SEM images and energy dispersive spectrum (EDS) were taken on an FEI Quanta 450 FEG scanning electron microscope equipped with an energy dispersive spectrometer. TEM images and selected area electron diffraction (SAED) analysis were carried out on a Philips CM200 field-emission transmission electron microscope (FE-TEM).

## Additional Information

**How to cite this article**: Liu, Y. *et al.* Copper nanocoils synthesized through solvothermal method. *Sci. Rep.* **5**, 16879; doi: 10.1038/srep16879 (2015).

## Supplementary Material

Supplementary Information
