# Supplementary material for: Copper nanocoils synthesized through solvothermal method
Source: Sci Rep. 2015 Nov 26;5:16879. doi: 10.1038/srep16879 (PMC4660362; doi:10.1038/srep16879)
Supplement: Supplementary Information [file srep16879-s1.pdf]

Supplementary information

Copper nanocoils synthesized through solvothermal method

Yanjuan Liu[1,2,+], Xiaowei Liu[3,4,+], Yongjie Zhan[1,*], Haiming Fan[2, §], Yang Lu[3,4, ¶]

1 Institute of Photonics and Photon Technology, Northwest University, Xi'an, Shaanxi, 710069, China

2 Department of Bioengineering, School of Chemical engineering, Northwest University, Xi'an, Shaanxi, 710069, China

3 Department of Mechanical and Biomedical Engineering, City University of Hong Kong, Kowloon, Hong Kong SAR, China

4 Center of Super-Diamond and Advanced Films (COSDAF), City University of Hong Kong, Kowloon, Hong Kong SAR, China

* corresponding author, E-mail: yjzhan@nwu.edu.cn

§ corresponding author, E-mail: fanhm@nwu.edu.cn

¶ corresponding author, E-mail: yanglu@cityu.edu.hk

[+] These authors contributed equally to this work.

1. **Suitable depositing and preserving of copper sample and destructive result in general washing process.**

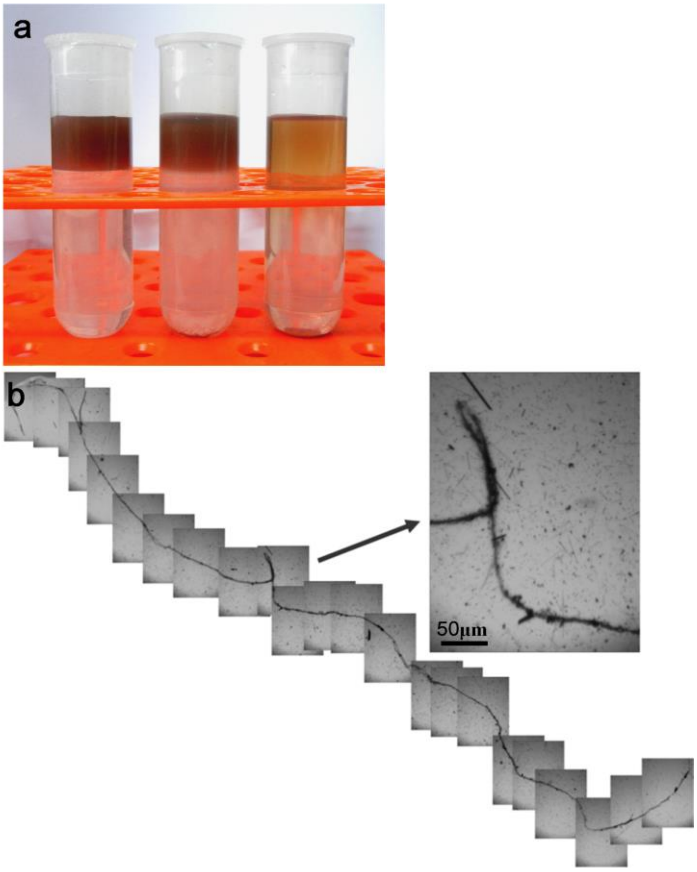

Figure. S1. Suitable and unsuitable treatments of copper samples. (**a**) Stock solutions (top layers) and NaCl solutions (bottom layers) before, in and after the suggested depositing treatment (in test tubes from left to right). Copper samples can be found on bottoms of latter test tubes (pointed by black arrows); (**b**) A strand of twisted copper nanowires and nanocoils formed in general washing and cleaning process.

General washing and cleaning operation using distilled water and various alcohols will introduce great disturbance and inevitably damage as-prepared nanostructures by intensely twisted them into "knitting wool", which is result of turbulent flow and is driven by density (of salts and polymer) gradients in mixed solutions. This universal effect did not affect normal nanowires seriously in most occasions, supersonic oscillating can unravel the "knitting wool" of nanowires almost nondestructively. However it may affect suspended nanocoils and nanorings seriously, and possibly damage them.

The hydrazine in preserving copper nanocoils:

The corrosion of copper in moisture circumstance follows such reaction function:

$$2Cu + O_2+H_2O+CO_2 = Cu_2(OH)_2CO_3 \quad (1)$$

We believe that the effect of hydrazine in keeping the copper sample is not reducing $Cu_2(OH)_2CO_3$ to elemental Cu, but simply blocking the oxidation path from Cu to Cu^{2+} in water before the formation $Cu_2(OH)_2CO_3$.

The choosing of hydrazine is based on results of series of control experiments. The hydrazine cannot totally prevent these copper nanostructures from corrosion, but it indeed greatly reduced the corrosion rate.

Without the protection of hydrazine, copper samples deposited on bottom of normal test-tube could be corroded within short period (such as a half day). However, with the protection of hydrazine, most of our copper nanocoils can still keep their circular shape in water even after 1-2 months, which is enough for most research purposes.

For SEM and TEM observations, the samples should be carefully washed with distilled water and the hydrazine shall be washed away. The dry state and vacuum environment, instead, can preserve the nanocoils from oxidation/corrosion, so in our SEM and TEM characterizations, interference of hydrazine can be essentially ruled out.

2. Diameter distribution of nanocoils

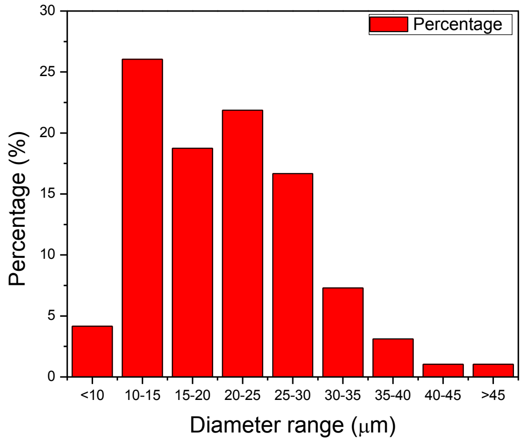

Figure. S2. Diameter distribution of nanocoils.

Statistics analysis is based on measurements of ~100 nanocoils recorded in our micrographs, in which samples were dispersed on copper grids or glass substrates for SEM/TEM//optical imaging. We believe that in untreated stock solutions, more nanocoils can be found, because sample cleaning and transferring processes will inevitably damage many nanocoils. From data shown, ring diameters of nanocoils are mainly in the range of 10-35μm.

3. **Evidences of end-closed nanocoils**.

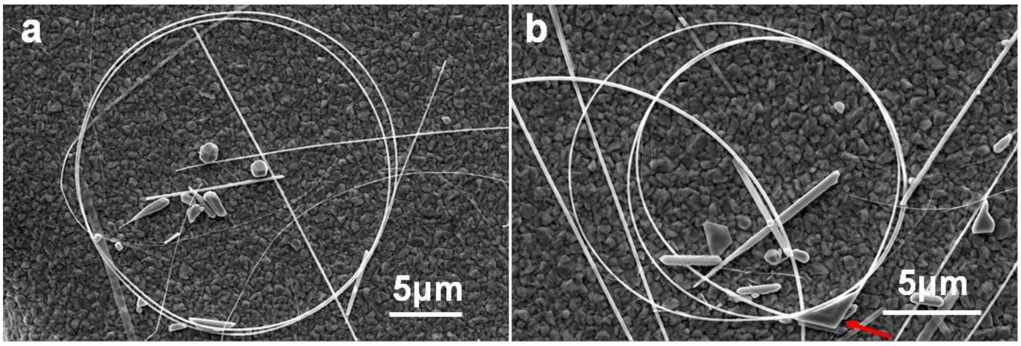

Figure S3. SEM images of end-closed multi-turns nanocoils. The jointing of their ends could be smooth (a) and unsmooth with an edge (b). Unsmooth jointing usually grew up to form a flaky structure.

4. Bent nanobelts

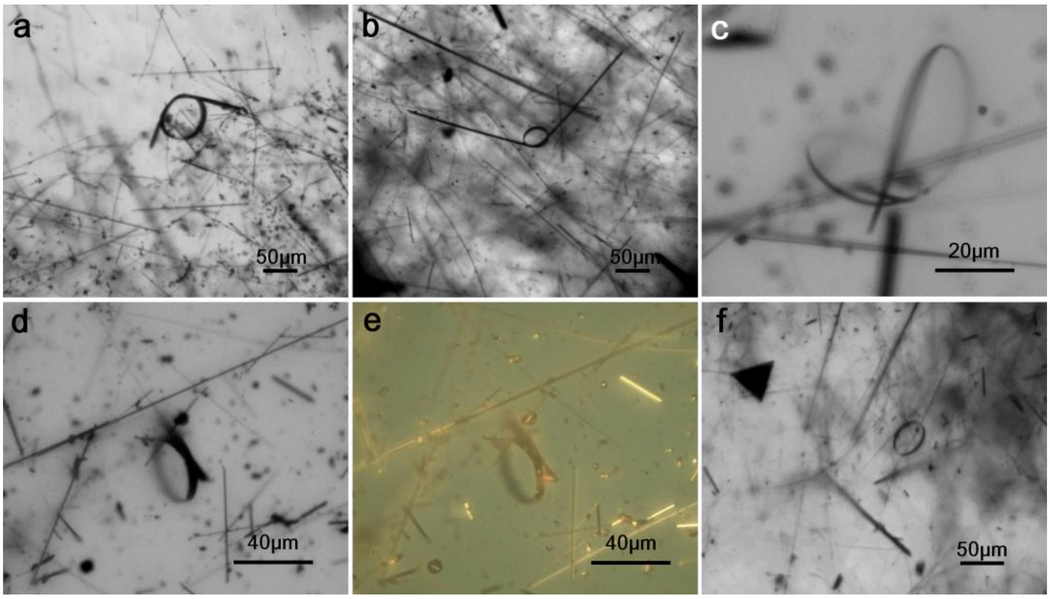

Figure S4. Optical images of bent nanobelts. (**a**, **b**, **d**, **e**) Nanobelts having spiral central sections and straight ends; (**c**) Nanocoil in self-interweaving; (**f**) one standard end-closed nanobelt. In (**d**) and (**e**) one same sample was recorded in transmitted light receiving mode and reflected light receiving mode respectively.

Nanobelts with circular midpieces are another common bent structure in stock solution. Their widths make a multi-turns structure rather difficult. These unclosed structures show stability to mild external disturbance, the bent midpieces and straight ends might suggest the different residual inner stresses.

5. Non-uniform broadening in growth from nanowires to nanobelts.

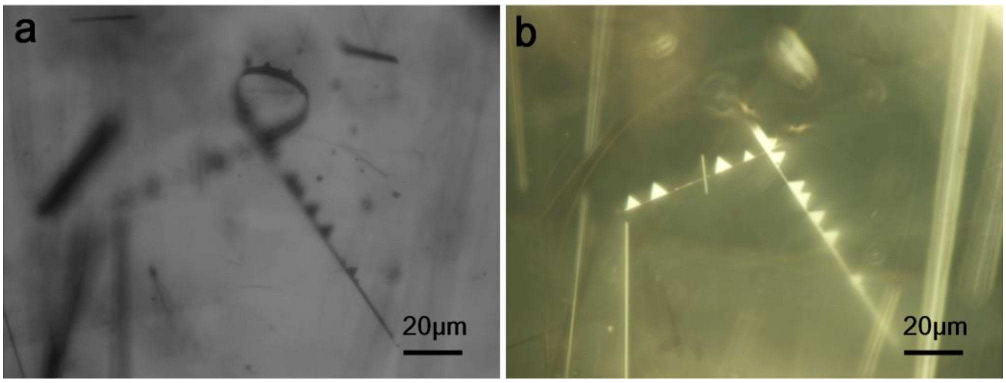

Figure S5. Optical images of one bent nanowire/nanobelt with a non-uniform edge.

A transition stage from nanowires to nanobelts. Triangle plates along the nanowires reveal that the surfaces of some nanobelts should be {111} crystal faces.
